# Supplementary material for: Patterns of Emergency Room Visits for Respiratory Diseases in New York State in Relation to Air Pollution, Poverty and Smoking
Source: Int J Environ Res Public Health. 2023 Feb 13;20(4):3267. doi: 10.3390/ijerph20043267 (PMC9966596; doi:10.3390/ijerph20043267)
Supplement: Supplementary file 1 [file ijerph-20-03267-s001.zip › ijerph-2165177-supplementary.pdf]

Supplement

Table S1. The Association between air pollution and respiratory diseases adjusted for poverty.

Respiratory Diseases	Term	Estimate	Standard error	Statistic	p-value
**Asthma**	(Intercept)	-3.342	0.053	-63.103	5.32E-53
	Air Pollution	0.137	0.031	4.398	5.05E-05
	Poverty	0.314	0.041	7.656	3.17E-10
**COPD**	(Intercept)	-3.364	0.063	-53.033	6.49E-49
	Air Pollution	-0.366	0.143	-2.549	0.01361
	Poverty	0.184	0.064	2.866	0.00588
**Acute Lower Respiratory**	(Intercept)	-3.433	0.087	-39.459	4.95E-42
	Air Pollution	-0.461	0.214	-2.153	0.03569
	Poverty	0.259	0.086	2.996	0.00410
**Acute Upper Respiratory**	(Intercept)	-2.886	0.064	-44.952	4.72E-45
	Air Pollution	0.014	0.056	0.254	0.80026
	Poverty	0.288	0.055	5.272	2.34E-06

Table S2. The Association between air pollution and respiratory diseases adjusted for smoking.

Respiratory Diseases	Term	Estimate	Standard error	Statistic	p-value
**Asthma**	(Intercept)	-3.293	0.079	-41.629	2.86E-43
	Air Pollution	0.158	0.051	3.082	0.00321
	Smoking	-0.110	0.086	-1.286	0.20399
**COPD**	(Intercept)	-3.353	0.061	-54.660	1.27E-49
	Air Pollution	-0.135	0.112	-1.203	0.23405
	Smoking	0.194	0.066	2.929	0.00494
**Acute Lower Respiratory**	(Intercept)	-3.399	0.083	-40.829	8.05E-43
	Air Pollution	-0.165	0.162	-1.024	0.31026
	Smoking	0.172	0.090	1.917	0.06039
**Acute Upper Respiratory**	(Intercept)	-2.844	0.077	-36.761	2.11E-40
	Air Pollution	0.098	0.067	1.464	0.14879
	Smoking	0.044	0.085	0.518	0.60678

Table S3. Rate of ER visits for respiratory disease, rate of smoking, rate of poverty at the county level.

FIPS	County	Asthma	COPD	Acute Lower	Acute Upper	Smoking	Poverty
**36001**	Albany	0.048	0.0213	0.0248	0.06	12.95	12.78889
**36003**	Allegany	0.0313	0.0479	0.0608	0.1239	20.7	17.45556
**36005**	Bronx	0.1944	0.0176	0.0234	0.1561	12.1	29.71111
**36007**	Broome	0.0377	0.0413	0.0303	0.0344	21.25	17.24444
**36009**	Cattaraugus	0.0242	0.0346	0.0427	0.0441	23.7	16.93333
**36011**	Cayuga	0.028	0.0267	0.0422	0.0364	19.65	13.02222
**36013**	Chautauqua	0.0399	0.0458	0.0593	0.0501	23.35	18.76667

**36015**	Chemung	0.0445	0.05	0.0681	0.0849	24.25	15.72222
**36017**	Chenango	0.0395	0.0668	0.0821	0.102	18	15.4
**36019**	Clinton	0.0522	0.0463	0.0567	0.0706	22.3	15.22222
**36021**	Columbia	0.0301	0.0259	0.0497	0.0424	17.55	11.64444
**36023**	Cortland	0.0353	0.0951	0.0382	0.1467	19.05	15.7
**36025**	Delaware	0.0302	0.0518	0.0414	0.0678	20.1	16.15556
**36027**	Dutchess	0.0346	0.0212	0.0307	0.0313	13.4	9.633333
**36029**	Erie	0.0414	0.024	0.0328	0.0554	17.05	14.66667
**36031**	Essex	0.0356	0.069	0.042	0.0665	16.6	12.51111
**36033**	Franklin	0.0243	0.0291	0.0253	0.033	24.7	18.72222
**36035**	Fulton	0.0498	0.0657	0.0628	0.1138	21.2	15.84444
**36037**	Genesee	0.0213	0.0411	0.0143	0.0301	22.45	12.06667
**36039**	Greene	0.0179	0.017	0.0246	0.0212	16.7	14.6
**36041**	Hamilton	0.0164	0.0218	0.0147	0.0228	13.3	10.58889
**36043**	Herkimer	0.0228	0.0418	0.029	0.0556	23.9	15.04444
**36045**	Jefferson	0.037	0.0378	0.1141	0.0983	22.55	14.95556
**36047**	Kings	0.1024	0.0184	0.018	0.0941	12.6	22.11111
**36049**	Lewis	0.0218	0.0407	0.0481	0.0663	16.35	13.81111
**36051**	Livingston	0.0228	0.0256	0.0224	0.0286	18.5	13.48889
**36053**	Madison	0.031	0.0426	0.0425	0.048	21.05	11.98889
**36055**	Monroe	0.0477	0.0235	0.0109	0.053	14.65	14.94444
**36057**	Montgomery	0.0796	0.0679	0.0943	0.1697	21.35	18.2
**36059**	Nassau	0.0292	0.0165	0.0081	0.0277	7.85	6.388889
**36061**	New York	0.0958	0.0155	0.0135	0.0776	8.8	17.35556
**36063**	Niagara	0.0366	0.0457	0.0427	0.0394	22.6	13.6
**36065**	Oneida	0.0363	0.0417	0.0402	0.0563	15.5	16.57778
**36067**	Onondaga	0.0327	0.0231	0.0166	0.0557	14.6	14.74444
**36069**	Ontario	0.0237	0.0409	0.0226	0.0403	19.65	10.06667
**36071**	Orange	0.0494	0.0283	0.0219	0.0407	12.1	12.43333
**36073**	Orleans	0.0309	0.0498	0.0144	0.0493	27.95	14.75556
**36075**	Oswego	0.0246	0.0407	0.0179	0.0385	25.5	17.56667
**36077**	Otsego	0.0178	0.0223	0.0319	0.0358	18.1	15.48889
**36079**	Putnam	0.0225	0.0151	0.0222	0.0224	12.35	5.988889
**36081**	Queens	0.0586	0.0161	0.015	0.0868	10.4	14.35556
**36083**	Rensselaer	0.0317	0.0252	0.0251	0.0534	18	12.25556
**36085**	Richmond	0.0559	0.0204	0.0136	0.0685	13.95	12.81111
**36087**	Rockland	0.0245	0.0088	0.0242	0.0232	7.3	14.01111
**36089**	St. Lawrence	0.0495	0.0659	0.0915	0.0876	16.45	18.46667
**36091**	Saratoga	0.0156	0.0159	0.0166	0.0173	14.65	6.822222
**36093**	Schenectady	0.0566	0.0439	0.0273	0.0839	14.85	12.6
**36095**	Schoharie	0.0145	0.0236	0.0132	0.0281	18.4	13.42222
**36097**	Schuyler	0.0345	0.0803	0.0542	0.0834	17.95	14.31111
**36099**	Seneca	0.0206	0.0347	0.0359	0.0458	17.4	13.3
**36101**	Steuben	0.0318	0.0514	0.0377	0.064	21.25	14.94444

**36103**	Suffolk	0.0338	0.021	0.0152	0.0333	14.5	7.311111
**36105**	Sullivan	0.0466	0.0536	0.0321	0.0765	19.7	17.34444
**36107**	Tioga	0.0107	0.0155	0.0113	0.0136	18.9	10.94444
**36109**	Tompkins	0.0162	0.0276	0.0122	0.0322	14.65	18.75556
**36111**	Ulster	0.0343	0.0309	0.0327	0.0432	14.3	13.65556
**36113**	Warren	0.0262	0.0326	0.0179	0.0227	21.15	11.55556
**36115**	Washington	0.024	0.0317	0.0189	0.025	23.8	12.95556
**36117**	Wayne	0.0273	0.0357	0.0172	0.0388	23.4	11.55556
**36119**	Westchester	0.0477	0.0178	0.0208	0.0487	7.7	9.6
**36121**	Wyoming	0.018	0.0295	0.0356	0.0428	22.3	11.35556
**36123**	Yates	0.0218	0.0479	0.0494	0.0634	11.05	14.83333

Table S4. chemical pollution per square mile at the county level.

FIPS	County	$PM_{2.5}$	VOCs	SO_2	NO_2	CO	Lead	Acetaldehyde	Formaldehyde	Benzene	Ethyl Benzene	Toluene	Xylene	Air Pollution
**36001**	Albany	11.94992	65.32933	32.62513	55.0205	206.4599	0.885912	0.69274	1.132095	0.543492	0.266922	2.636021	0.985205	126.1757
**36003**	Allegany	1.986771	26.51794	0.233439	4.166388	24.24236	0.050355	0.410362	0.59309	0.08994	0.039425	0.344034	0.138082	19.60406
**36005**	Bronx	85.53523	937.8707	48.50371	584.3502	2544.893	2.554315	3.509507	5.961198	6.808656	4.619518	71.43836	15.57565	1437.207
**36007**	Broome	4.99875	42.11458	1.716079	16.76837	95.44779	0.195317	0.557304	0.802639	0.29457	0.130614	1.167499	0.492789	54.89544
**36009**	Cattaraugus	2.122795	26.83426	0.344732	5.073692	30.15631	0.170108	0.398265	0.561591	0.116592	0.049121	0.37102	0.189996	22.12949
**36011**	Cayuga	4.610048	27.41182	1.294824	11.34604	52.80376	0.626246	0.439009	0.624519	0.201853	0.096277	0.753921	0.402499	33.53694
**36013**	Chautauqua	3.554722	38.63314	7.654646	16.04342	56.85168	0.213096	0.457884	0.681157	0.235123	0.09929	0.824832	0.422531	41.89051
**36015**	Chemung	5.59261	45.73311	3.827869	17.87258	78.7358	0.22932	0.550055	0.78435	0.293875	0.120959	1.106516	0.499639	51.78223
**36017**	Chenango	2.316759	24.23961	0.353966	3.997102	28.09195	0.125442	0.427896	0.59296	0.106649	0.042174	0.459432	0.182193	20.31205
**36019**	Clinton	3.064568	36.69639	0.613811	6.457449	46.86274	0.057983	0.597787	0.836635	0.176131	0.080061	0.615063	0.352268	32.13696
**36021**	Columbia	3.536523	43.31418	0.690688	8.157116	53.86789	0.185389	0.543661	0.781841	0.195475	0.097009	0.726249	0.361191	37.48574
**36023**	Cortland	2.916325	26.4683	0.473641	7.74621	42.9065	0.202311	0.44772	0.631656	0.139782	0.077123	0.689362	0.274642	27.65786
**36025**	Delaware	1.503862	21.32606	0.419187	3.350195	22.55153	0.061029	0.363897	0.506466	0.077283	0.041644	0.373764	0.161091	16.912
**36027**	Dutchess	6.458236	56.73755	2.223373	16.19709	123.3523	0.927848	0.580454	0.834318	0.387418	0.176804	1.502983	0.675435	70.01792
**36029**	Erie	10.40868	71.49228	12.70453	49.02188	250.8579	1.547552	0.686704	1.088414	0.713593	0.359019	3.539388	1.333438	134.5845
**36031**	Essex	1.374287	32.25188	1.749527	3.990656	27.62334	0.131365	0.565686	0.777806	0.095385	0.049125	0.382196	0.260309	23.08385
**36033**	Franklin	1.321265	28.37189	0.246215	2.486264	23.35829	0.045031	0.537672	0.739539	0.08584	0.045578	0.308804	0.19758	19.24799
**36035**	Fulton	3.287939	38.12657	0.660747	5.378811	53.84035	0.183934	0.624978	0.885778	0.217153	0.094046	0.808571	0.396446	34.83511
**36037**	Genesee	5.977865	29.8395	0.646283	17.01006	65.69034	0.611808	0.460837	0.679578	0.217454	0.100942	0.851357	0.372276	40.81943
**36039**	Greene	3.165817	38.57998	0.692068	9.287579	52.95636	0.064769	0.523542	0.739434	0.16373	0.087238	0.654983	0.320476	35.74533
**36041**	Hamilton	0.334478	25.99456	0.030727	0.96562	24.08742	0.016955	0.448202	0.619674	0.100581	0.105324	0.753246	0.40653	17.95444
**36043**	Herkimer	1.667897	22.87338	0.251226	5.111688	27.03819	0.05814	0.440518	0.648335	0.097626	0.047237	0.359191	0.185487	19.59297
**36045**	Jefferson	3.438748	31.67576	1.112995	10.97161	56.19931	0.385709	0.524253	0.750304	0.200342	0.096772	0.718775	0.433167	35.50258
**36047**	Kings	106.5534	1026.68	37.46419	707.7738	3121.149	3.475724	3.991314	7.55731	8.138398	5.316534	78.27231	17.51305	1707.962
**36049**	Lewis	1.391078	23.47399	0.160841	2.681611	19.98304	0.042014	0.44781	0.618469	0.071499	0.042889	0.34461	0.156325	16.47139
**36051**	Livingston	4.314558	29.06255	0.437779	8.378578	44.99818	0.479744	0.453763	0.641309	0.163749	0.07832	0.661193	0.313035	29.99425
**36053**	Madison	3.742624	26.01638	0.515127	8.083432	45.92101	0.195521	0.469404	0.659904	0.162225	0.071526	0.622583	0.2663	28.90868
**36055**	Monroe	13.73117	80.1524	40.17351	63.55693	350.1834	1.059542	0.759231	1.175334	0.930027	0.504789	4.44976	1.836461	186.1709
**36057**	Montgomery	4.473489	33.26958	0.803751	17.31371	62.63838	0.089289	0.516313	0.751993	0.197683	0.107636	0.964625	0.424638	40.51703

**36059**	Nassau	29.72829	263.0826	13.36672	208.8948	1308.83	0.539279	1.801181	2.88439	3.168317	1.891578	17.53755	7.034075	619.5863
**36061**	New York	470.7446	2597.658	360.7866	3324.868	13663.42	20.20104	13.06751	31.23945	26.24584	16.59369	189.122	55.41906	6923.123
**36063**	Niagara	7.848136	48.54728	37.02858	47.35074	129.7074	0.480349	0.594167	0.931749	0.418313	0.203348	2.082551	0.789256	91.99395
**36065**	Oneida	4.708924	32.66449	1.229064	11.46869	70.17546	0.181627	0.510775	0.732572	0.245583	0.101747	0.894512	0.394128	41.10253
**36067**	Onondaga	9.124555	55.13749	5.706816	39.29832	208.1945	0.293396	0.612014	1.001117	0.549853	0.27567	2.524832	1.069672	107.9294
**36071**	Orange	6.79929	65.85844	9.381628	26.63823	147.6719	1.253163	0.591074	0.877933	0.43326	0.192979	2.116725	0.755183	87.52325
**36073**	Orleans	4.294081	28.52026	0.996183	11.14973	47.63506	0.115027	0.507719	0.642468	0.188923	0.105829	0.89446	0.384061	31.81127
**36075**	Oswego	4.254474	34.50703	1.438857	10.26626	63.78867	0.140759	0.537322	0.764849	0.242371	0.102636	0.831775	0.457477	39.11083
**36077**	Otsego	2.320079	23.9873	0.438195	4.927762	29.71097	0.131685	0.409021	0.571013	0.107153	0.048653	0.397589	0.180632	21.07669
**36079**	Putnam	6.809704	90.18017	2.31996	21.77784	173.5531	0.045255	0.825787	1.196359	0.4917	0.257679	2.021362	0.995069	100.158
**36081**	Queens	84.34814	730.6642	50.13331	698.3427	2721.683	3.480457	4.155604	8.625861	6.813275	4.179289	52.77456	14.71389	1459.971
**36083**	Rensselaer	4.680948	43.85086	1.196462	11.81096	75.45166	0.047084	0.549531	0.78017	0.26128	0.118006	1.117863	0.431124	46.76531
**36085**	Richmond	41.19471	355.759	12.64148	325.8013	1412.356	0.594272	2.356093	3.849414	3.841922	2.281533	23.82819	8.217338	730.907
**36089**	St. Lawrence	2.022193	25.89127	3.158615	4.566981	46.38145	0.080452	0.481246	0.666273	0.097803	0.041544	0.310216	0.169898	27.95598
**36091**	Saratoga	6.786922	52.78347	1.44636	16.15137	113.8996	0.406199	0.651382	0.954689	0.388763	0.16003	1.516111	0.595195	65.2467
**36093**	Schenectady	9.028524	80.45117	3.72074	36.00623	200.4479	1.330192	0.863662	1.297168	0.620504	0.312361	2.882708	1.196689	112.7193
**36095**	Schoharie	2.387931	28.56195	0.340387	4.431188	31.3672	0.029793	0.439119	0.68809	0.121166	0.06855	0.575929	0.24357	23.08496
**36097**	Schuyler	3.8047	42.51973	3.091967	6.668997	53.62061	0.056993	0.545718	0.766457	0.205287	0.163781	1.325702	0.592152	37.78736
**36099**	Seneca	4.902634	43.53626	0.943342	12.48934	77.83846	0.224675	0.521539	0.955503	0.314447	0.199708	1.381032	0.87994	48.06229
**36101**	Steuben	3.10637	26.93547	0.328898	8.005375	34.4845	0.131586	0.395419	0.573351	0.126353	0.058408	0.479122	0.238274	24.95438
**36103**	Suffolk	15.81804	139.7674	13.64282	97.79169	609.6947	3.334802	1.160152	1.80717	1.529524	0.898864	7.665043	3.476452	298.8622
**36107**	Tioga	3.561482	33.3903	0.510915	8.354199	49.38121	0.055233	0.506716	0.715664	0.17167	0.08177	0.682244	0.298225	32.56988
**36109**	Tompkins	5.239125	35.78274	30.55293	17.769	68.18707	0.475145	0.48643	0.70484	0.247967	0.112465	1.030783	0.437592	53.67536
**36111**	Ulster	3.489851	46.14419	1.481324	9.241442	64.80865	0.187107	0.514602	0.726652	0.225116	0.100621	0.813966	0.389402	42.70764
**36113**	Warren	3.195028	43.35392	1.9278	13.19156	61.6556	0.296339	0.652316	0.926612	0.215322	0.134604	0.869195	0.556146	42.32481
**36115**	Washington	3.606114	33.49228	0.634408	6.029938	36.69362	0.320557	0.519206	0.742223	0.144784	0.051995	0.479839	0.199298	27.63809
**36117**	Wayne	4.91768	32.40395	0.851714	12.81982	63.76334	0.268952	0.470211	0.669232	0.235004	0.104167	1.024023	0.412962	39.31369
**36119**	Westchester	18.03242	151.7631	7.665015	97.87284	691.8091	1.642161	1.104453	1.693971	1.646846	0.940244	8.4499	3.594955	328.7383
**36123**	Yates	3.96398	38.3014	0.569074	7.335977	55.69003	0.329419	0.517228	0.727567	0.232128	0.16632	1.282376	0.625745	36.58042
